# A physical description of the adhesion and aggregation of platelets

**DOI:** 10.1098/rsos.170219

**Published:** 2017-04-12

**Authors:** Bastien Chopard, Daniel Ribeiro de Sousa, Jonas Lätt, Lampros Mountrakis, Frank Dubois, Catherine Yourassowsky, Pierre Van Antwerpen, Omer Eker, Luc Vanhamme, David Perez-Morga, Guy Courbebaisse, Eric Lorenz, Alfons G. Hoekstra, Karim Zouaoui Boudjeltia

**Affiliations:** 1 Comupter Science Department, University of Geneva, CUI, 7 route de Drize, 1227 Carouge, Switzerland; 2 Laboratory of Experimental Medicine (ULB 222 Unit), Université Libre de Bruxelles (ULB), CHU de Charleroi, Belgium; 3 Microgravity Research Centre, Université Libre de Bruxelles (ULB), Belgium; 4 Laboratory of Pharmaceutical Chemistry and Analytic Platform of the Faculty of Pharmacy, Université Libre de Bruxelles (ULB), Belgium; 5 Institute of Molecular Biology and Medicine, Université Libre de Bruxelles (ULB), Belgium; 6 Department of Interventional Neuroradiology, CHRU de Montpellier, France; 7 CREATIS, INSA Lyon, University of Lyon, Lyon, France; 8 Computational Science Laboratory, University of Amsterdam, Amsterdam, The Netherlands; 9 ITMO University, Saint Petersburg, Russia

**Keywords:** mathematical model, platelet deposition, adhesion and aggregation rates, shear-induced diffusion, whole blood *in vitro* experiments

## Abstract

The early stages of clot formation in blood vessels involve platelet adhesion–aggregation. Although these mechanisms have been extensively studied, gaps in their understanding still persist. We have performed detailed *in vitro* experiments, using the well-known Impact-R device, and developed a numerical model to better describe and understand this phenomenon. Unlike previous studies, we took into account the differential role of pre-activated and non-activated platelets, as well as the three-dimensional nature of the aggregation process. Our investigation reveals that blood albumin is a major parameter limiting platelet aggregate formation in our experiment. Simulations are in very good agreement with observations and provide quantitative estimates of the adhesion and aggregation rates that are hard to measure experimentally. They also provide a value of the effective diffusion of platelets in blood subject to the shear rate produced by the Impact-R.

## 1. Introduction

The current literature gives a quite fractionate picture of platelet adhesion (process by which they bind to a vessel wall) and aggregation (process by which they attach to each other). Both experimentally and numerically, sub-processes have been considered separately, disregarding the fact that several of them actually coexist and compete. The resulting emergent behaviour of the whole system may differ significantly from that produced by the constitutive elements taken in isolation.

Owing to the importance of platelet deposition in clinical practice and physiology [1], it is essential to provide a quantitative description, including all the relevant elements present in real conditions. Medical treatments require a complete understanding of the phenomena involved in physiology and pathology. A precise physical description will weigh the role of various molecules and allow scientists to identify new therapeutic targets, not only on the adhesion and aggregation but also on the spreading of platelets which is much less known in clinical practice. Indeed, the visualization of this process is poorly documented and rather challenging. Barr *et al.* [2] reported interesting pictures showing the platelet spreading on mice carotid sub-endothelium. In addition, several studies have associated alterations of the spreading *in vitro* with *in vivo* modifications of thrombi formation [3,4].

To characterize platelet deposition to a new quantitative level, we used an approach in which *in vitro* experiment and numerical simulations are developed in synergy.

Many papers explore the platelet adhesion–aggregation process by numerical simulations [5]. However, these research efforts do not take into account the different roles of pre-activated and non-activated platelets in bloodstream from donors [6,7], the possible inhibition processes [8–10], the counting of platelets and the three-dimensional structure of each aggregate.

Here, we revisit this question with *in vitro* experiments in a closed system, using the platelet function analyser Impact-R [11] combined with a new original method to determine the three-dimensional structure of platelet aggregates. We interpret the experimental observations in terms of a numerical model implementing first principle processes, and whose parameters can be deduced from the measurements. The co-development of the experiment and model turned out to be instrumental to discover the new results presented in this study. Of course, it is worth noting that our results are obtained within a given experimental setting. Although we believe that they are qualitatively correct in general, this remains to be further investigated.

## 2. Experimental set-up

The Impact-R is a cylindrical device whose lower end is a fixed disc, serving as a deposition surface, with a total area *S*=132.7 mm^2^. It is covered with polystyrene, on which platelets adhere and aggregate. Other coatings can also be considered to study, for instance, the deposition of platelets on collagen.

The upper end of the Impact-R cylinder is a rotating disc, shaped as a cone with a small angle of 2.45^°^. Both the upper and lower discs are aligned with the *xy*-plane. Anticoagulated (citrate) samples of whole blood were loaded between these two discs, separated by *L*=0.82 mm. Owing to the rotation of the upper disc, the blood is subject to a laminar flow. A controlled shear rate at the wall $\dot{\gamma }$ is created in a given observation window of 1×1 mm^2^, on the deposition surface. We imposed $\dot{\gamma }=100\;s^{-1}$, corresponding to a value where the platelet deposition reaches its maximum in a range of 0–5000 s^−1^ (see electronic supplementary material, figure S1). It also corresponds to the order of magnitude for which thrombosis is observed to occur in cerebral aneurysms [12,13]. Note also that for such a low shear rate, we may expect that the secondary flow effects described in [14] are not present, and that the von Willebrand factor can be neglected [8,15]. By performing experiments with different adhesion mediators (data not shown), we observed that the integrin IIbIIIa is the receptor predominantly involved for adhesion and aggregation at the proposed shear rate, in agreement with [8,15].

**Figure 1.**
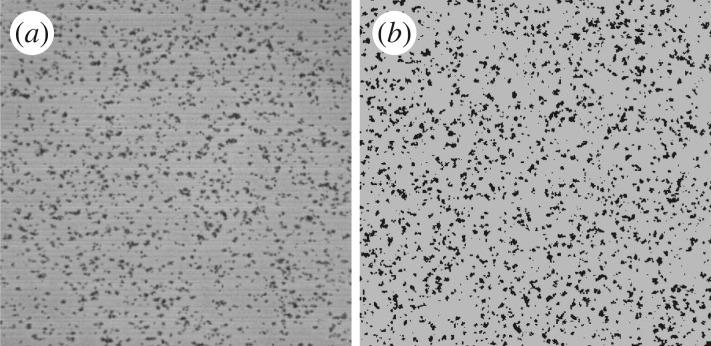
(*a*) Platelet deposition as observed on the Impact-R 1×1 mm deposition window, after 300 s. (*b*) Result of the numerical simulation, obtained with the parameters of table 1.

The experiment was repeated for seven healthy donors. For each of them, we measured, in the observation window, the formation of clusters resulting from deposition and aggregation of platelets (figure 1*a*). Note that the same deposition pattern is also observed at other locations over the substrate, indicating a homogeneous behaviour in the rotation plane.

The number of clusters and their size, as well as the number of activated platelets and non-activated platelets still in suspension were measured at 20 s, 60 s, 120 s and 300 s. For each of these times, a new experiment had to be performed, because the measurement requires the interruption of the deposition process.

## 3. Observations

Our goal is to explain the observed time evolution of the above quantities as a function of the parameters of the system. Numerous mathematical models have been proposed in the literature to describe the adhesion of platelets on a surface. See for instance [16,17] and references therein. All the models assume that platelets reach the deposition surface due to a shear induced diffusion. The other parts of the proposed models depend very much on the specific question addressed by the authors, and the experimental device they have considered to produce their observations (often assuming a steady state, which is not the case here). Our first attempts to model the Impact-R experiments convinced us that the current knowledge is not sufficient to explain the observed data.

An unexpected feature was the fact that platelet deposition is limited by some unidentified mechanism. During the first 60 s, we observe a rapid increase of the number of clusters but, at 300 s, while there still exists a large number of platelets in suspension, no new formation of clusters is observed, and the cluster surfaces do not increase any longer.

**Figure 2.**
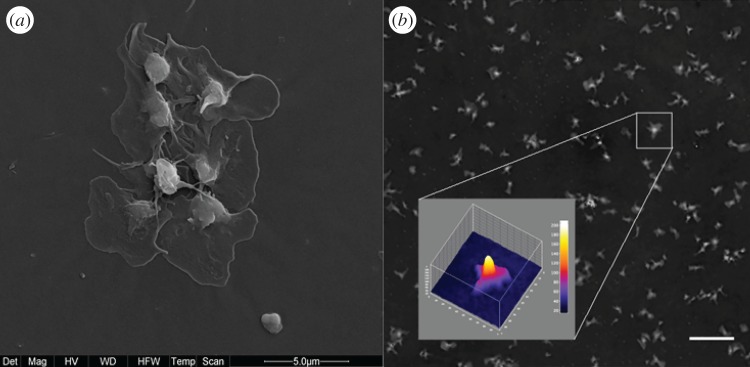
(*a*) Scanning electron microscopy image (magnification 12 000×) of platelet aggregates in the well. (*b*) The three-dimensional shape of a platelet aggregate based on the optical height obtained by digital holographic microscopy. The vertical scale bar unit is 5.2 nm and the field of view 12.8 ×12.8 μm. Scale bar, 20 μm.

We first attempted to explain the slowdown of the increase of cluster areas by assuming a dominant growth in the third dimension (*z*-axis). Using a digital holographic microscope, we were able to study the development of the aggregate thickness [18], a quantity never measured before to our knowledge (figure 2). However, the three-dimensional nature of the aggregate was not sufficient to explain the saturation of the areas of the clusters. We performed new experiments (see electronic supplementary material, figures S3 and S4) that revealed that blood albumin is the main factor limiting adhesion and aggregation, as it competes with platelets for deposition. This is compatible with the results reported in [19], in a different experimental setting.

To account for the above observation, we propose the following scenario. (i) Activated platelets (AP) adhere to the deposition surface, thus forming a seed for a new cluster. (ii) Non-activated platelets (NAP) and AP can deposit at the periphery, or on top, of an existing cluster. This is the aggregation mechanism that makes the cluster grow. (iii) Albumin (Al) also deposits on the surface, thus reducing locally the adhesion and aggregation rates of platelets. Note that, due to the thrombogenic property of polystyrene, we do not expect platelets to detach once deposited.

## 4. Mathematical model

To describe and validate the above process, we propose the following numerical model. The substrate *S* is aligned with the *xy*-plane and the blood layer expands along the *z*-axis. We consider three types of particles: AP, NAP and Al. Any of them can be either in suspension in the blood layer, or deposited on *S*. We consider a mixed particle-density representation: particles in suspension are described by a density field but as individual entities when they deposit.

The blood layer has thickness *L*=0.82 mm and is subject to a constant shear rate $\dot{\gamma }=100\;s^{-1}$. As reported in various studies (e.g. [20]), this shear rate will affect the motion of the red blood cells (RBC) in suspension, and create the so-called shear-induced diffusion of platelets and albumin towards the deposition substrate.

The deposition substrate *S* is discretized as *n*×*n* square cells of area Δ*S*=5 (μm)^2^, corresponding to the size of a deposited platelet (obtained as the smallest variation of cluster area observed with the microscope). The number *n* is chosen as $1000\;\text{μ}m/\sqrt{5\;{\left(\text{μ}m\right)}^{2}}=447$ so that the total area of the substrate is *S*=1 mm×1 mm, as in the experiment.

Assuming a good horizontal mixing in the *xy*-plane due to the rotating flow, we consider that the densities of AP, NAP and Al only vary along the *z*-axis and can be described by three one-dimensional diffusion systems from which we will determine the probability that AP, NAP or Al deposits on each cell of the substrate. This assumption considerably speeds up the computation as we replace the three-dimensional bulk dynamics by a one-dimensional model. As a result the exploration of the parameter space will be done in an acceptable time.

Accordingly, we assume that the AP, NAP and Al particles in the bulk are described by

4.1 $$\partial_{t}\rho =D\partial_{z}^{2}\rho \;J=-D\;\mathrm{grad}\;\rho ,$$

where *ρ* is the density of either AP, NAP or Al, *J* is the flux of particles and *D* the shear induced diffusion.

The initial densities *ρ*(*z*)=*ρ*_0_ are independent of *z*, and are determined by experiment: 172 200 (μl)^−1^ for NAP, 4808 (μl)^−1^ for AP and 2.69×10^13^ (μl)^−1^ for Al.

The boundary condition at the top of the blood layer is *J*(*L*,*t*)=0 at any time. On the deposition layer, we first define a boundary layer of thickness Δ_*z*_. It describes the region just above the substrate where particles are available for deposition. Let us denote by *N*(*t*) the average number of particles in a volume Δ*S*×Δ_*z*_ in the boundary layer. Thus, the boundary condition for the density of suspended AP, NAP and Al at *z*=0 is *ρ*(0,*t*)Δ*S*Δ_*z*_=*N*(*t*).

The boundary layer is populated by the particles reaching the substrate by diffusion from the bulk, and depleted by the deposition on the substrate. This is described by the following equation for *N*(*t*):

4.2 $$\dot{N}=-J(0,t)\Delta S-p_{d}N(t),$$

where *p*_d_ is the deposition rate, which will evolve during time and will vary across the substrate, according to the deposition history. In order to implement this process, particles are considered as individual entities that can deposit on any cell (*i*,*j*) of the substrate.

Let *δt* be the time discretization and *p*_d_(*i*,*j*,*t*) the deposition rate on cell (*i*,*j*) at time *t*. Then, *p*_d_(*i*,*j*,*t*)*N*(*t*)*δt* is interpreted as the probability that a new platelet deposits on that cell. The total number *m*(*t*) of newly deposited particles on the entire substrate is therefore

4.3 $$m(t)=\sum_{ij}[\mathrm{rand}(i,j)<p_{d}(i,j,t)N(t)\delta t],$$

where rand(*i*,*j*) are random numbers uniformly distributed in [0,1[. The term *p*_d_*N*(*t*) in equation (4.2) is then computed as *m*/(*n*^2^*δt*), i.e. the average number of deposited particles per cell and per time unit. Note that for albumin, *p*_d_(*i*,*j*,*t*)*N*(*t*)*δt* is directly taken as the number of newly deposited molecules.

The deposition rates *p*_d_(*i*,*j*,*t*) are implemented as follows. On cells already occupied by a platelet, a new platelet can only deposit on top, thus increasing the thickness of the aggregate. This rate is noted *p*_top_ and is supposed constant over time. On platelet-free cells, a new platelet can deposit at a rate which is different for AP and NAP and depends on the amount of albumin already in this cell. NAP can only deposit in a cell next to an existing aggregate. Al can only deposit in a platelet-free cell.

The exact deposition rules are the following. An albumin that reaches the substrate at time *t* deposits with a probability *P*(*t*) which depends on the local density *ρ*_al_(*t*) of already deposited Al. We assume that *P* is proportional to the remaining free space in the cell,

4.4 $$P(t)=p_{\mathrm{al}}(\rho_{\max}-\rho_{\mathrm{al}}(t)),$$

where *p*_al_ is a parameter and $\rho_{\max}$ is determined by the constraint that at most 100 000 albumin particles can fit in the area Δ*S*.

An activated platelet that hits a platelet-free cell on *S* deposits with a probability *Q*, where *Q* decreases as the local concentration *ρ*_al_ of albumin increases. We assumed that

4.5 $$Q=p_{\mathrm{adh}}\exp(-\lambda \rho_{\mathrm{al}}),$$

where *p*_adh_ and λ are parameters. This expression can be justified by the fact that a platelet needs more free space than an albumin to attach to the substrate, due to their size difference. In other words, the probability of having enough space for a platelet decreases roughly exponentially with the density of albumin in the substrate. This can be validated with a simple deposition model on a grid, where small and large objects compete for deposition.

Once an activated platelet has deposited, it is the seed of a new cluster that grows further due to the aggregation of further platelets. In our model, AP and NAP can deposit next to already deposited platelets. From the above discussion, the aggregation probability *R* is assumed to be

**Figure 3.**
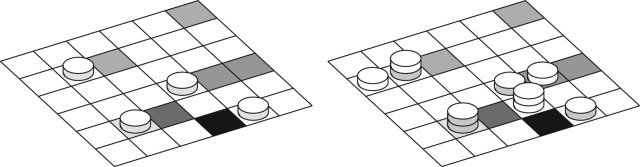
Sketch of the deposition substrate, discretized in cells of area Δ*S*, corresponding to the surface of a platelet. The grey levels indicate the density of albumin already deposited in each cell. The picture also illustrates the adhesion, aggregation and deposition along the *z*-axis. On the left panel, activated platelets (grey side discs) deposit first. Then in the right panel, non-activated platelets (white side discs) aggregate next to an already formed cluster.

4.6 $$R=p_{\mathrm{agg}}\exp(-\lambda \rho_{\mathrm{al}}),$$

with *p*_agg_ another parameter. Figure 3 illustrates the proposed deposition rules.

The above model can be simulated numerically with different values of the unknown parameters (*p*_al_, *p*_adh_, λ, *p*_agg_ and *p*_top_), in order to reproduce the time evolution of the *in vitro* experiment, namely the number *N*_c_(*t*) of clusters per mm^2^, the average cluster size *s*(*t*) and the number *N*_ap_(*t*) and *N*_nap_(*t*) of platelets still in suspension (activated and non-activated, respectively).

However, the shear-induced diffusion coefficient *D* and the thickness of the boundary layer Δ_*z*_ need to be determined. The value of *D* from the Zydney–Colton theory (e.g. [16,17,21]) produces a flux of platelets towards the deposition substrate with *D*≈5×10^−11^ m^2^ s^−1^ for the direction perpendicular to the flow, when considering $\dot{\gamma }=100\;s^{-1}$ and a hematocrit of 40%. This value is however too small to explain the 3125 platelets per microlitre that have disappeared on average from the bulk within the first 20 s of the experiment, even with the maximum deposition rate. In a column of section Δ*S*=5 (μm)^2^ and height *L*=0.82 mm (i.e. 4.1 μl), this amounts to about 12 800 deposited platelets.

**Figure 4.**
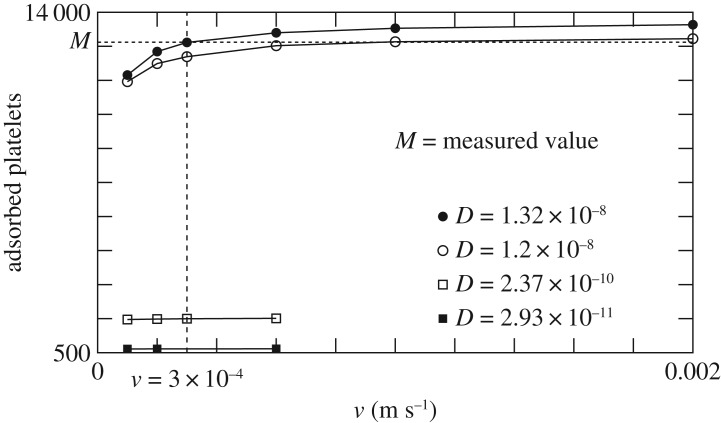
Number of particles adsorbed after 20 s, in the random walk model.

To determine *D*, we take the smallest value compatible with the observed depletion of platelets from the bulk within the first 20 s, assuming that all platelets hitting the surface adhere. This problem is solved with a particle-based diffusion–deposition model. The experimentally determined number of particles in a column of section Δ*S*=5 (μm)^2^ and height *L*=0.82 mm are randomly distributed in space. Then they undergo a discrete-time (but continuous-space) random walk: at every time step Δ*t* each particle randomly changes its velocity to ±*v* and moves accordingly. Particles that cross the line *z*=0 are removed. Those reaching *z*=*L* are bounced back from were they came. Using *D*=*v*^2^Δ*t*/2, we can choose Δ*t* as a function of *D* and explore the deposition count after 20 s for different *v* and *D*, as illustrated in figure 4.

**Figure 5.**
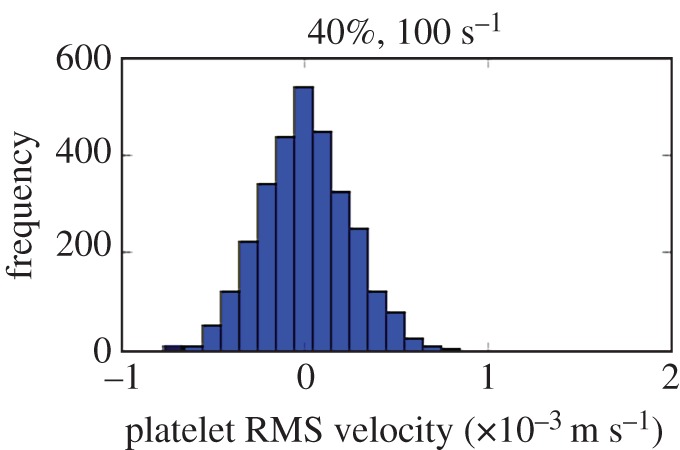
Platelet velocity distribution in a two-dimensional blood shear flow, with fully resolved RBC and platelets.

The typical value of *v* is based on two-dimensional simulations of a bloodflow subject to a shear rate of 100 s^−1^ and hematocrit of 40%, with fully resolved RBC and platelets (see [22,23] for details of the model used for these simulations). The measured velocity distribution of platelets is shown in figure 5 and the order of magnitude of *v* is chosen as the standard deviation.

From figure 4, we conclude that to match the experiment, assuming a perfect adsorption, the diffusion coefficient *D* is required to be *D*≥1.2×10^−8^. The published value *D*≈5×10^−11^ m^2^ s^−1^ for $\dot{\gamma }=100\;s^{-1}$ and hematocrit of 40% is clearly too small to explain the observed deposition rate, independently of a precise choice of *v*.

In the following, we chose *D*=1.3×10^−8^ m^2^ s^−1^, corresponding to the molecular velocity *v*=3×10^−4^ m s^−1^, as obtained from the fully resolved simulation of platelets and RBC shown in figure 5. Possible explanations for this unexpected value of the diffusion coefficient will be further discussed below.

The discrete-time, continuous-space random-walk model can also be used to determine Δ_*z*_, the thickness of boundary layer that is needed to properly implement the diffusion–deposition process. We varied Δ_*z*_ in the system of equations (4.1) and (4.2) so as to match the density profile with that of the random-walk model, with several values of deposition probabilities. These numerical investigations show that Δ_*z*_=2×10^−5^ m produces a good agreement between the continuous and discrete models.

## 5. Results and discussion

The above model for adhesion and aggregation of platelets, including the presence of albumin, has been run for 5 min of physical time, with *D*=1.3×10^−8^ m^2^ s^−1^ and Δ_*z*_=2×10^−5^ m. We checked the independence of the result with respect to spatial and temporal discretization of the one-dimensional diffusion models, which is not obeyed if the boundary layer Δ_*z*_ is omitted.

**Figure 6.**
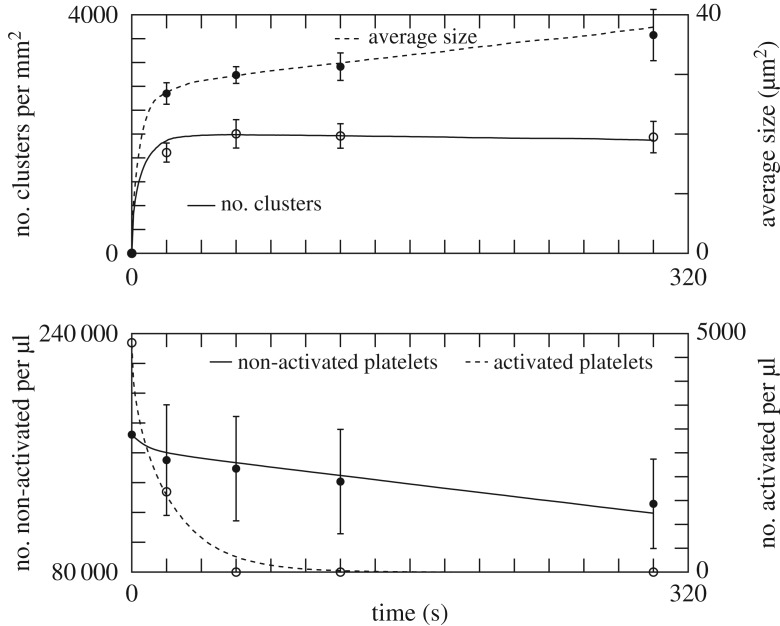
The result of the adhesion and aggregation model (continuous and dashed lines) and the experimental data (points). The parameters used in the simulation are given in table 1.

**Figure 7.**
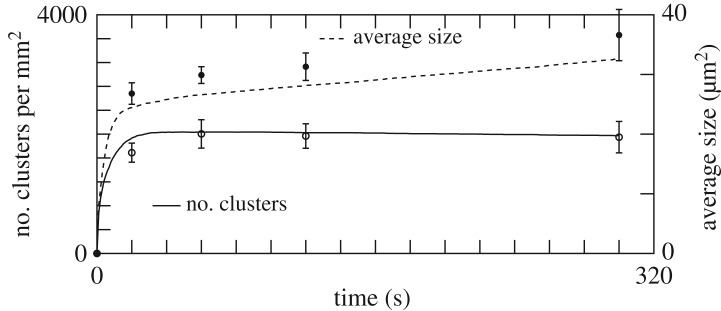
Same as figure 6, but with *p*_add_=120 s^−1^ and *p*_agg_= 14 s^−1^. The size of the aggregates is very sensitive to this reduction of *p*_agg_. The curves describing the time evolution of the number of pre-activated or non-activated platelets are very similar to those of figure 6, and not repeated here.

Table 1 reports the values of *p*_al_, *p*_adh_, λ, *p*_agg_ and *p*_top_, obtained by exploring parameter space, that produce an optimal agreement with experiment, as depicted in figure 6. Changing the value of these parameters affects the quality of the results, as illustrated in figure 7. The effect on the deposition pattern of removing albumin is illustrated in the electronic supplementary material, figure S6.

**Table 1.** The parameters of the model found to provide the best fit of the experimental data (figure 6), for $\dot{\gamma }=100\;s^{-1}$ and a polystyrene coating.

*p*_adh_ (s^−1^)	*p*_agg_ (s^−1^)	*p*_al_ (s^−1^)	*p*_top_ (s^−1^)	λ (μm^2^)
110	14.6	1.7×10^−3^	0.6	30

The high value of *p*_adh_ suggests that adhesion is diffusion-limited, whereas aggregation is reaction-limited. Figure 1*b* shows the simulated deposition pattern after 5 min. Figure 6 shows that our model reproduces quantitatively the *in vitro* measurements, thus confirming the proposed scenario of deposition and aggregation, and the competition with albumin. Note that the values in table 1 are expected to depend on the shear rate $\dot{\gamma }$ and the nature of the substrate. Such a dependence was indeed observed by Shenkman *et al.* [24] when changing the composition of the deposition surface with, for instance, a fibrinogen or a von Willebrand factor (vWF) coating.

In our experimental set-up, several substrates were used to coat the polystyrene surface. The use of extracellular matrix (laminin, fibronectin, collagen IV) interfered with the number of platelet aggregates formed and their average size, but without altering the qualitative behaviour (see electronic supplementary material, figure S7). We performed also complementary experiments to address the role of major platelet receptors, such as GPIb and IIb/IIIa, in our experimental conditions (see electronic supplementary material, figure 8). As expected, the Impact-R experiments confirmed the dominant role of receptor IIb/IIIa at low shear rate and of GPIb at high shear rate. The role of blood vWF was found to be important only at high shear rate (see electronic supplementary material, figure 9). Moreover, we also observed that our model responds to the adenosine diphosphate supplementation (see electronic supplementary material, figure 10). This is in agreement with a previous work reported in [25].

**Figure 8.**
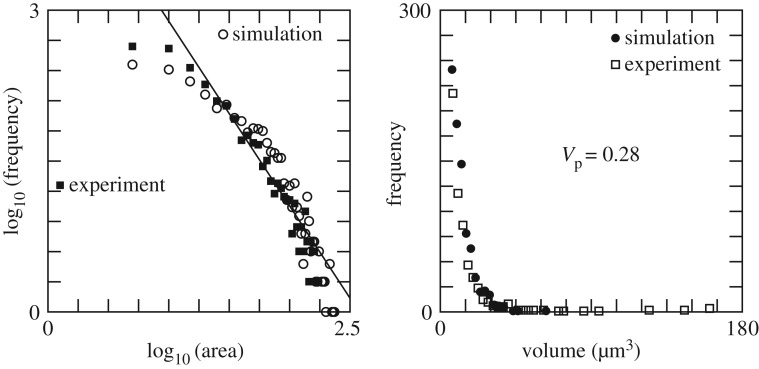
(*a*) Distribution of the size of clusters at *t*=300 *s*. The area is given in square micrometres. (*b*) Distribution of cluster volumes at *t*= 300 *s*. The data suggest a power law with exponent −2.8. Simulation parameters are given in table 1.

To further test the model against the experiment, we compared the distribution of sizes *s* and volumes of the clusters measured by digital holographic microscopy (figure 8). The size distribution from the simulation matches very well that of the experiment, at 20 s (data not shown) and 300 s. The log–log plot suggests a power law distribution *p*(*s*,*t*)=*α*(*t*)*s*^−*β*(*t*)^, with *β*(20 s)=2.23 and *β*(300 *s*)=1.84.

From the simulation, one obtains the distribution of the number of particles per cluster, including those that piled up along the *z*-axis. Assigning a volume of *V*_p_=0.28 (μm)^3^ to each aggregated platelet leads to a volume distribution compatible with the *in vitro* measurements (figure 8*b*). This volume is smaller than the volume of a platelet in suspension (6 (μm)^3^) because, as indicated by observations with a holographic microscope [18], platelets lose a significant part of their volume during deposition. Our selected value of *V*_p_ also yields a match between the experimentally measured total volume *V*_tot_=1 701272 (μm)^3^ of deposited aggregates and the total numerical number *N*_tot_=6 032 000 of deposited platelets.

This study has revealed unexpected phenomena in the adhesion–aggregation processes of platelets, namely the competition with albumin and the different roles played by activated and non-activated platelets. By adhesion, activated platelets initiate a new cluster which mostly grows due to the non-activated platelets. By analysing the three-dimensional structures of the aggregates, we could obtain the distribution of areas and volumes. By tuning the model parameters so as to fit the *in vitro* time observations, the adhesion and aggregation rates can be measured. The excellent agreement between the model and the experiment gives a strong credit to the plausibility of the proposed scenario.

Our study has also revealed that the Zydney-Colton shear induced diffusion coefficient is significantly too small to explain the observed deposition rate. It is well known that the transport of RBC and platelets in a shear flow is a very complex phenomenon [20], and not yet fully understood. For instance, Kumar & Graham and Eckstein & Belgacem [20,26] report that the flux of platelets is better described with a drift-diffusion model *J*=−*D* grad *ρ*−*ρ* grad *Φ*, where *Φ* is called the ‘rheological potential’, describing the collision of platelets with an inhomogeneous distribution of RBC. However, the precise expression for *Φ* is still to be derived. We are currently working on this problem, using the fully resolved bloodflow simulations developed in [22,23]. Preliminary results in a two-dimensional simulation [27] have shown a very fast initial migration of the platelets towards the vessel wall, in a time scale incompatible with a pure diffusion process with Zydney–Colton diffusion coefficient. Interestingly however, the thickness of the platelet margination layer that forms next to the wall would be compatible with the experimentally observed flux of platelets assuming the Zydney–Colton diffusion coefficient.

With the current lack of a better understanding, it is interesting to notice that the effective diffusion coefficient we obtained in this study in order to explain the observed deposition rate gives a very good account of the dynamics of the whole deposition process.

Strictly speaking, the above results apply to the present experimental setting. But due to the general concepts we have used, both for the model and the experiment, we expect them to be qualitatively correct in other situations relevant to biomedical science.

## Supplementary Material

A physical description of paltelet deposition: supplementary data

## Data accessibility

Our C++ simulation code is available at http://cui.unige.ch/~chopard/RSOS/ together with explanations on how to run it. All the experimental data relevant to this work are presented in the paper or the electronic supplementary material.
