# Supplementary material for: A physical description of the adhesion and aggregation of platelets
Source: R Soc Open Sci. 2017 Apr 12;4(4):170219. doi: 10.1098/rsos.170219 (PMC5414280; doi:10.1098/rsos.170219)
Supplement: A physical description of paltelet deposition: supplementary data [file rsos170219supp1.pdf]

# A physical description of the adhesion and aggregation of platelets

## Supplementary information

## Determination of platelet adhesion and aggregation in function of the shear rate and time

Venous blood was drawn from a homogeneous group of healthy donors with ages between 25 and 65 years old into tubes with 3.2% sodium citrate solution, pH 7.4.

Platelet aggregates formation was induced by exposing 130 µL of whole blood in a well to a laminar flow produced by the Impact-R device. The device is programmed to produce laminar flows over a fixated polystyrene disk of 132.7 $mm^2$ by the rotation movement of a disposable Teflon conical rotor, parallel to the first disk, and placed at 0.82 mm, above it. Controlled wall shear rate is created in an observation window of 1 x 1 $mm^2$. After washing, images from a ring on the deposition plane were captured by the image analyzer on impact-R, which quantifies the platelet aggregates formed, in 2D, on the surface. The results are expressed as number of aggregates detected and the average size of the aggregates, according to the shear stress. This device can produce laminar flows in the range of 25 $s^{-1}$ to 5000 $s^{-1}$ and with exposition times between 20 and up to 300 seconds.

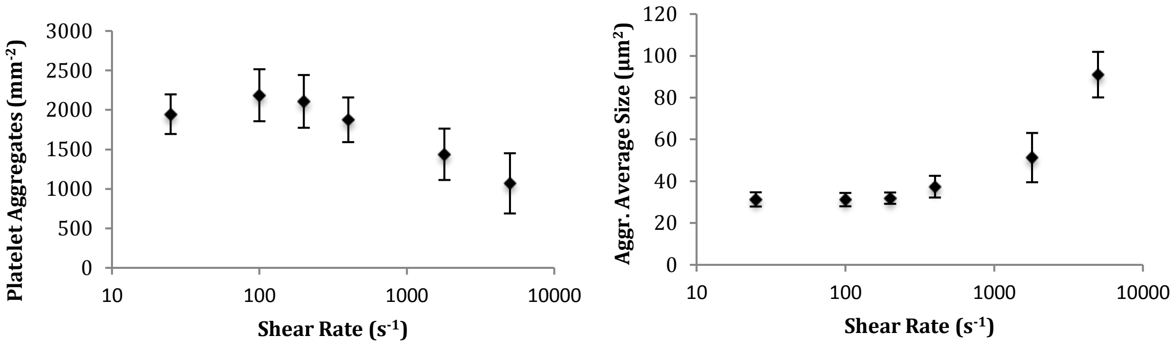


**Supplementary figure 1** – Number of platelet aggregates created in the 1 $mm^2$ observation window of the polystyrene surface when 130 µl samples of anticoagulated whole blood (citrated) are subjected to controlled laminar shear rates (25, 100, 200, 400, 1800 and 5000 $s^{-1}$) during 2 minutes at room temperature by the Impact-R platelet function analyzer (n=7).

## 3D analysis of platelet aggregates.

Platelet aggregates, created on the Impact-R wells polystyrene surfaces at different exposition time were analyzed using the Digital Holographic Microscope (DHM). For the present study, whole blood from 7 donors was exposed to a shear rate of 100 $s^{-1}$ (the experimental condition for which the number platelet aggregates is maximum- Suppl. Fig. 1).

The 3D analytical method was based on the analysis of 5 images in the same ring from the Impact-R wells, captured by the CCD camera associated to the DHM following, the published methodology (Biomed Opt Express. 2015 Sep 1; 6(9): 3556–3563. doi: 10.1364/BOE.6.003556).

A)

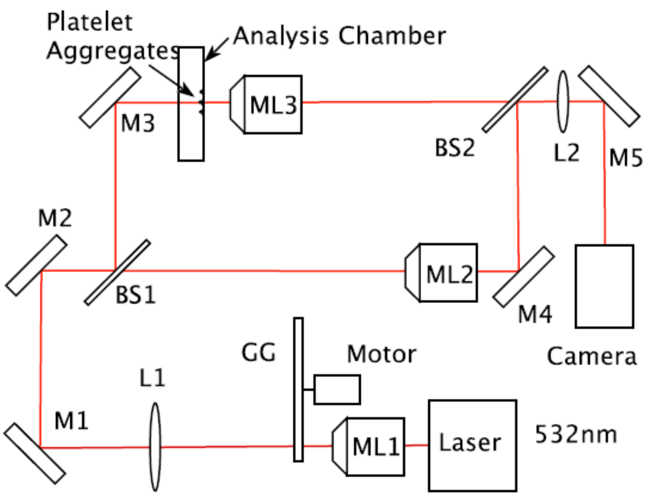


B)

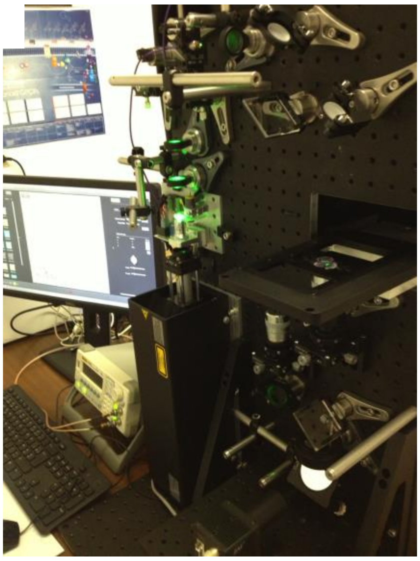

**Supplementary figure 2** – A) Digital holographic microscope schema: GG: Rotating Ground Glass; L1 and L2: Lenses; BS1 and BS2: Beam Splitters; ML1-3: Microscope Lenses, M1-5: Mirrors. B) Digital Holographic Microscope complete system.

## Total and pre-activated platelets count during the tests.

For these analysis, 20 µl of blood collected from the samples before and after the Impact-R test are analyzed by flow cytometry (SYSMEX® XN 1000). Experimental conditions under investigation: exposure of the whole blood samples to a laminar flow of 100 $s^{-1}$ for 5, 20, 60, 120 and 300 seconds. Before starting the test, a sample was also recovered and analyzed, to determine the original concentration of pre-activated and non activated platelets.

The number of activated platelets in suspension was determined by double detection using flow Cytometry. CD42P (BD Pharmingen: REF#558819) antibody was used to isolate blood platelets and activated platelets were detected using PAC-1 (BD Biosciences: REF#340507) and CD62P (APC: REF:550888) antibodies. Isotypes control antibodies were used as threshold to determine the activated platelets. PAC-1 recognizes an epitope on the glycoprotein IIb/IIIa complex of activated platelets and CD62P recognizes elements of the inner wall of α-granules that are exposed on the outside of platelets upon its activation.

## Albumin impact in thrombus formation

Serum albumin, the most abundant protein in human blood plasma, plays pivotal role in the transport of hormones, fatty acids and other compounds, it buffers pH and maintains osmotic pressure. Given that blood has a high concentration of this molecule (3-4 g/dL) we hypothesize that this molecule my also impact platelet interactions. In order to test the existence of a possible role of the protein portion of plasma in the mechanism of platelet adhesion and aggregation, the polystyrene surface from the Impact-R wells were treated with one of the following coating solutions: Serum, Plasma, Bovine Serum Albumin solution (BSA, mimicking the same concentration of albumin present in human serum) and Hank's Balanced Salt Solution (HBSS).

The coatings with serum or with BSA blocked the adhesion of platelets to the well, while no significant difference were observed with the plasma or HBSS coating. Thus, albumin plays a pivotal role in preventing platelets from adhering to the well.

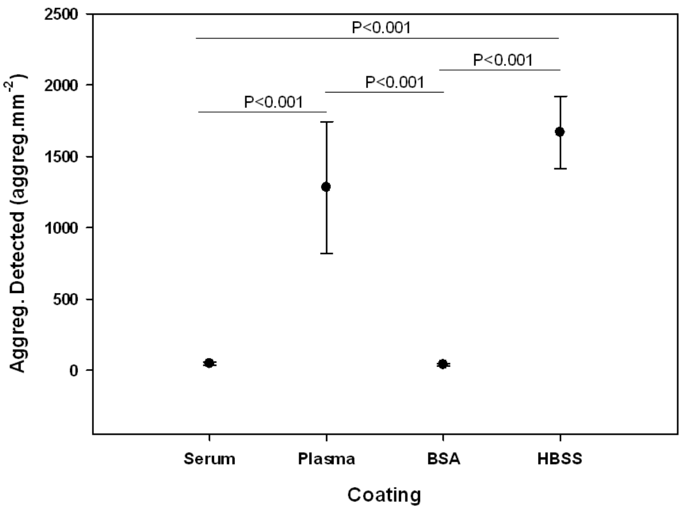


**Supplementary figure 3** – Number of platelet aggregates created when 130 µl of whole blood samples are deposited in Impact-R polystyrene wells coated with different solution: serum, plasma, BSA (3 g.dL$^{-1}$) and a control coating solution (HBSS) and exposed to a laminar shear rate of 100 s$^{-1}$ for 2 minutes.

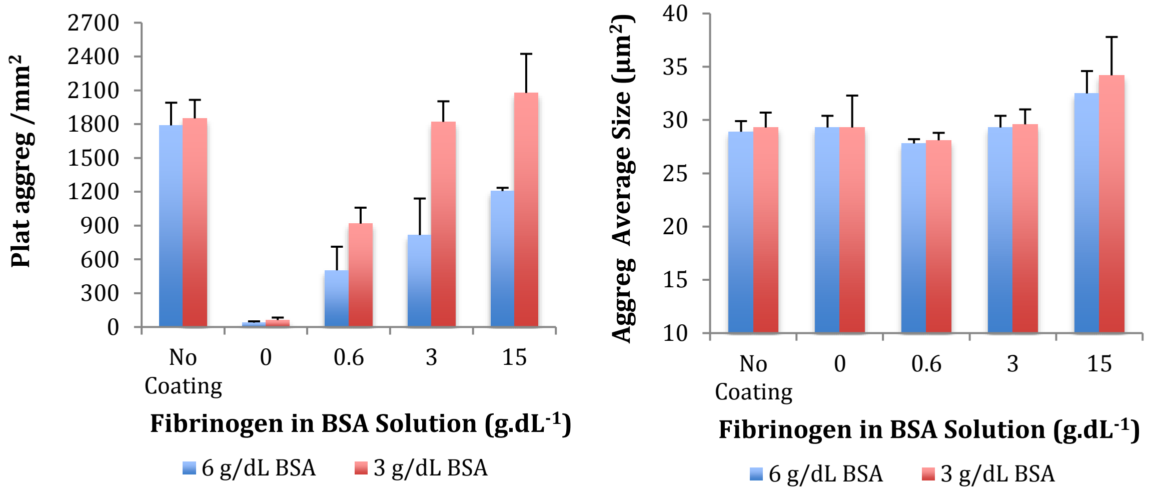


**Supplementary figure 4** - Impact of the coating of different mixed concentrations of fibrinogen and albumin on platelet adhesion and aggregation properties when citrated whole blood samples are exposed to a laminar flow with shear rate of 100 $s^{-1}$ for 120 seconds (n=5) Mean +/- SEM.

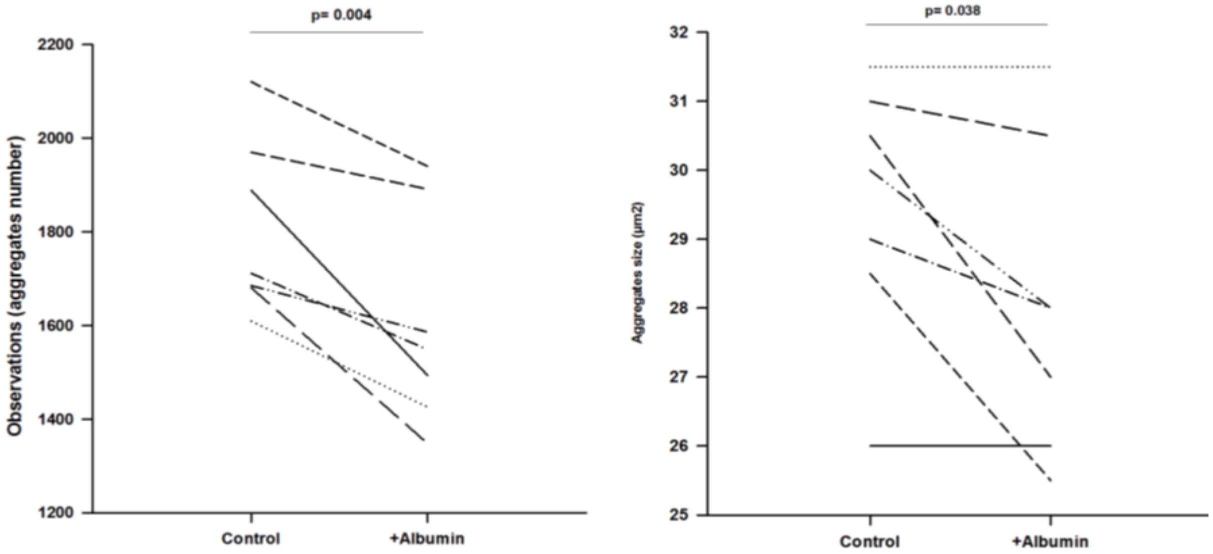


**Supplementary figure 5**- Number of platelet aggregates formed and their average size obtained by exposing whole blood samples with physiological and altered content in albumin of a group of 7 volunteers to a laminar flow of 100$s^{-1}$ for 120 seconds using Impact-R platelet function analyzer.

While the experimental observations demonstrate the role of albumin as a limiting factor for platelet adhesion aggregation, our numerical model is also very sensitive to the albumin concentration. This is illustrated in Suppl Fig 6 where we simulate a situation with a deficit of albumin. As a result, the aggregate size increase much faster.

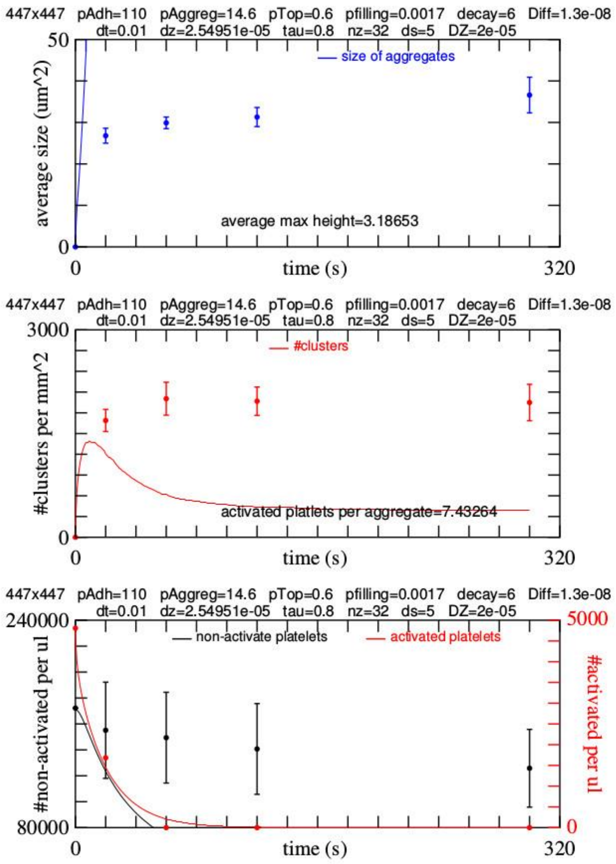


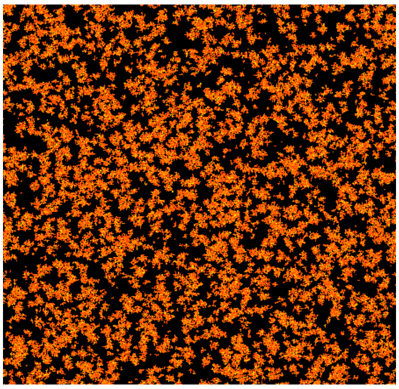

**Supplementary figure 6** – Experiments and simulations showed that the increase of the concentration of albumin resulted in a reduction of the number of platelet aggregates formed and an increase of the aggregates size in the Impact-R test. This figure shows the result of the simulation with 20% of the normal concentration of albumin. We see that the size of the aggregate grows much too fast, due to less competition with albumin.

Several substrates were used to coat the polystyrene surfaces. The use of extracellular matrix components such as laminin, fibronectin or collagen (IV) interfered with the number of platelet aggregates formed and their average size, but without altering the qualitative behavior. More aggregates are formed at lower shear rate magnitude and as the shear rate increased, the average size of the aggregates formed increases as well (see next suppl figure 7, where a summary of the results from this study is presented).

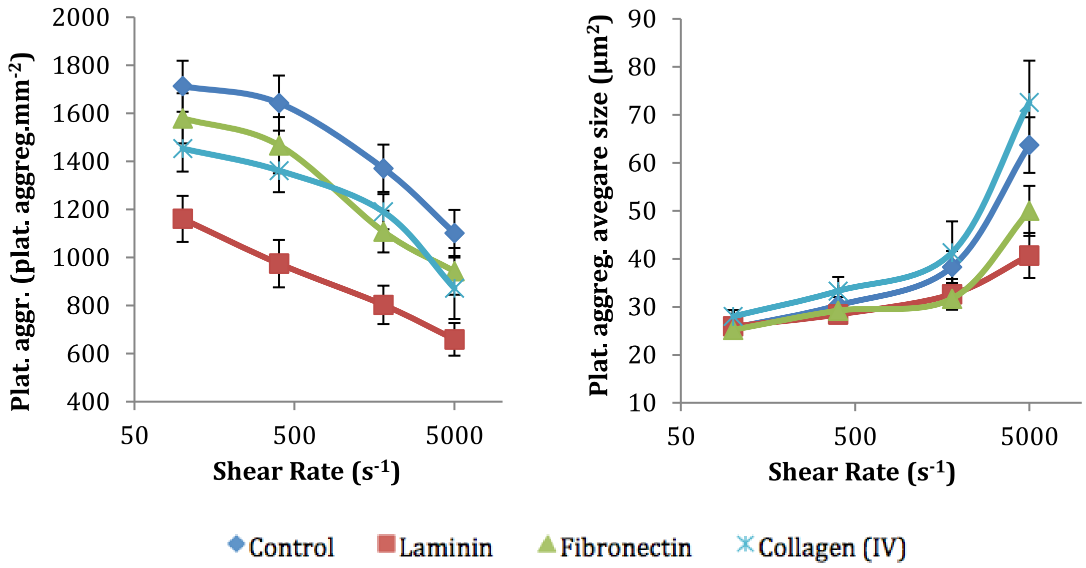


**Supplementary Fig 7** - Impact of the presence of different extracellular proteins molecules on platelet adhesion and aggregation properties studied under different hemodynamic conditions (n=18 for control, laminin and fibronectin coatings; for collagen n=6) 120 seconds, Mean +/- SEM.

In order to address the role of IIb/IIIa and GPIb receptors in our system, experiments were performed using two blockers. Results are presented bellow.

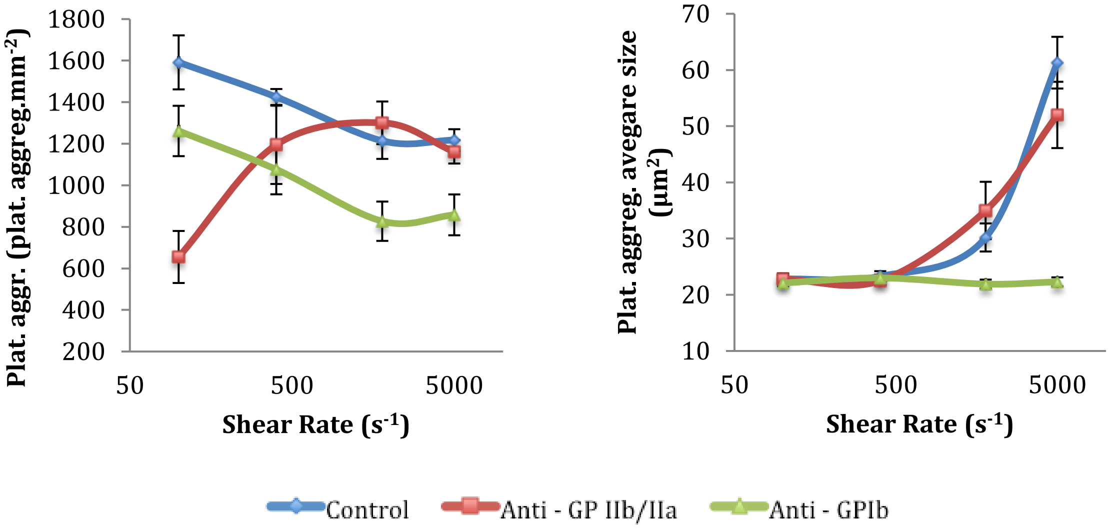


**Supplementary Figure 8** – Impact on the adhesion and aggregation properties of the blocking of the platelet receptors GPI and integrin IIbIIIa. (n=6), 120 seconds.

According to our results, the blocking of GP1b resulted in a significant decrease of the number of aggregates formed in all the shear rate conditions tested. Additionally, the blocking of GP1b also interfered with the aggregation properties of platelets since it prevented the aggregates to expand when blood samples were exposed to high shear rate conditions.

In what concerns the blocking of the integrin IIbIIIa, we observed a significant impact on the number of platelet aggregates formed at low shear rate conditions (100 and 400 $s^{-1}$), but no effect at higher shear rate. Thus, in our experimental set IIbIIIa receptor plays a predominant role in the processes of platelet adhesion and aggregation at the low range of shear rate.

Our results are in agreement with the study published by M. Maxwell (*Blood 2007 109:566-576*) that show that the GPIb receptor plays a predominant role at shear rates over 1000$s^{-1}$ in interaction with the vWF in the processes of platelet adhesion and that IIbIIIa plays a predominant role at lower shear rate magnitude.

We also addressed the role of <u>VWF and fibrinogen</u> amount in plasma from volunteers in the adhesion process.

The current understanding is that at low shear rate (0 à 1000 $s^{-1}$) platelet aggregation is primarily mediated by soluble fibrinogen, which physically crosslinks platelets through engagement of integrin αIIbβ3. At progressively higher shear rate (1000 $s^{-1}$ à 10000 $s^{-1}$) aggregation becomes increasingly driven by the von Willebrand Factor through its ability to rapidly engage glycoprotein Ib, with fibrinogen playing a supportive role in stabilizing aggregates, as reported by B. Savage et al (*Cell 1996 84:289 and Cell 1998 94:657*).

In what concerns vWF, in our experiments we confirm the link between the vWF in blood and the platelet adhesion at high shear rate. A good correlation is reported at 1800 $s^{-1}$ (R= 081; p<0.001). In contrast, no significant correlation with the plasma fibrinogen was observed for the experiments performed at 100, 400 or 5000 $s^{-1}$.

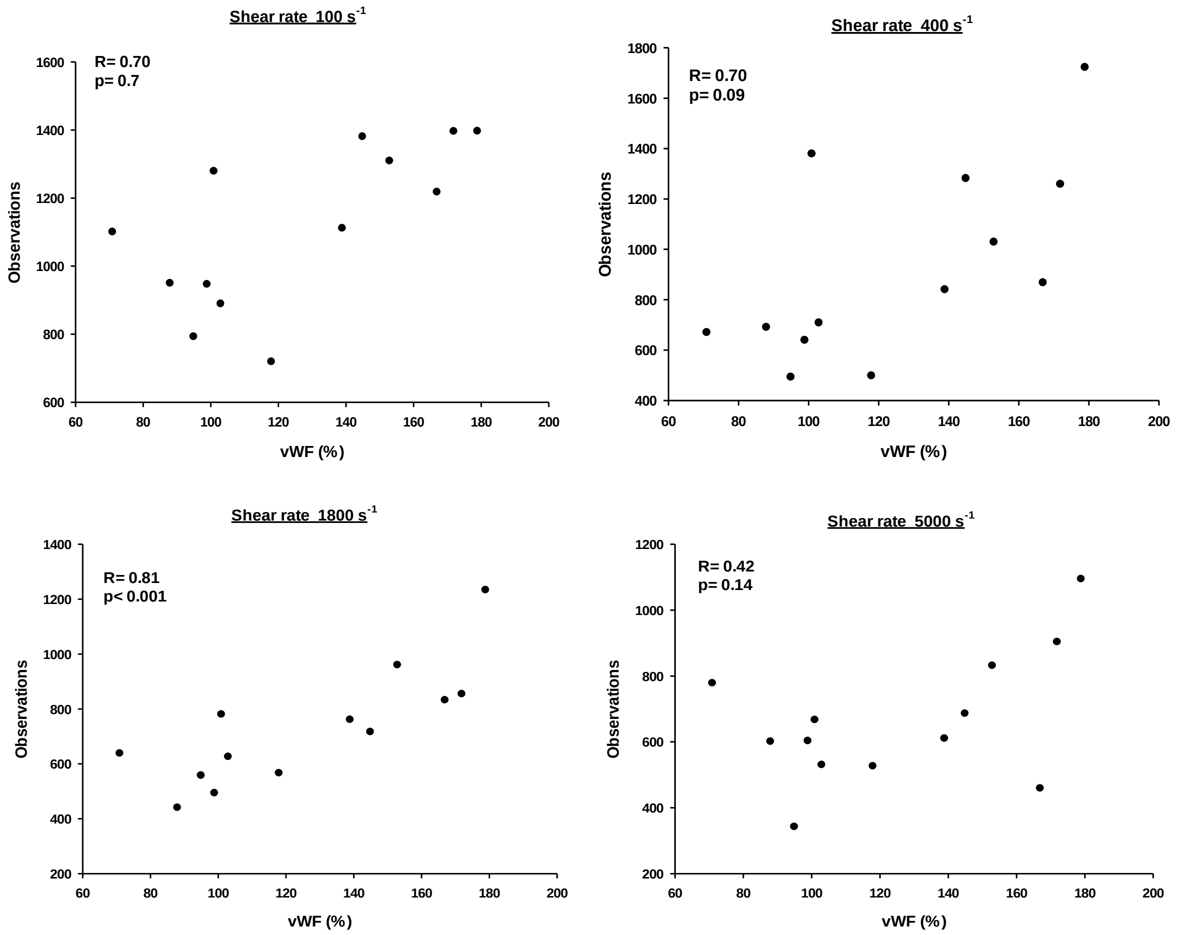


**Supplementary fig 9** – Correlation between the % of vWF (as compared to the referential value) from each subject and the number of platelet aggregates formed when citrated whole blood samples are exposed to a laminar flow with different shear rate magnitudes (n=13).

As we considered a shear stress of 100 $s^{-1}$ in our simulations, the effect of plasma vWF from the subject in the adhesion process was dismissed. This remark was added to the manuscript.

A set of experiments was performed in order to study the impact of the addition of external ADP or its antagonist prostaglandin E1 (PGR1) in the experimental system. These molecules were added to the blood sample and incubated for 1 minute under agitation before starting the test. Samples were exposed to a laminar flow with imposed shear rate of 100 $s^{-1}$. Two exposition times were tested: 20 and 120 seconds.

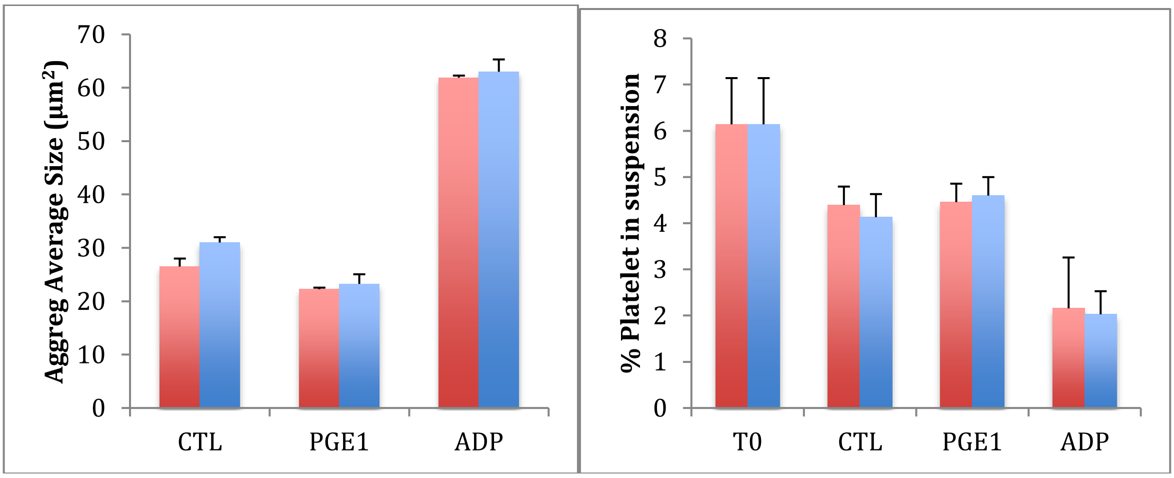


**Supplementary fig 10** - Impact of the addition of ADP (10µM final concentration) or ADP pathway antagonist PGE1 (1µM final concentration) on the aggregation (a) or the percentage in suspension (b) of platelets after the test when citrated whole blood samples were exposed to a laminar flow with shear rate of 100 $s^{-1}$ for 20 (red) or 120 seconds (blue). T0 corresponds to the concentration of platelets before starting the test (n=2) in tube just after the venipuncture.

Results indicate that the addition of external ADP, as expected, potentiates the aggregation of platelets. The aggregates formed on the polystyrene surface were fewer in number but significantly bigger (as compared to the control condition). Additionally, an important decrease in the number of platelets in suspension was observed in the sample following the test, which indicates that the addition of external ADP increased the reactivity of platelets.

Interestingly, an antagonist of the ADP pathway (PGE1) in our model does not changes the platelet in suspension and the aggregate sizes. This suggests that in our experimental setup the intrinsic ADP does not plays a critical role.
